# Successful use of the forced choice test for detecting concealment of semantic memory in criminal and intelligence investigations

**DOI:** 10.3389/fpsyg.2024.1399985

**Published:** 2024-06-07

**Authors:** Tzachi Ashkenazi, Gil Goldzweig, Aldert Vrij, Sharon Leal

**Affiliations:** ^1^Department of Criminology, Ashkelon Academic College, Ashkelon, Israel; ^2^School of Behavioral Sciences, The Academic College of Tel Aviv-Yaffo, Tel Aviv, Israel; ^3^Department of Criminology, Bar-Ilan University, Ramat Gan, Israel; ^4^Psychology Department, University of Portsmouth, Portsmouth, United Kingdom

**Keywords:** deception detection, concealed knowledge detection, forced choice test, semantic memory, terror organization, criminal investigations, intelligence investigations

## Abstract

The current study examined the validity of the forced choice test (FCT) in a forensic scenario when used to detect concealment of semantic memory (SM-FCT). We also compared the SM-FCT validity to the FCT validity in the more commonly investigated episodic memory scenario (EM-FCT). In simulating a scenario of investigating suspected members of a terror organization, 277 students were asked to deceptively deny being enrolled in a college in which they do actually study. Results indicated that the SM-FCT’s validity level was within the range of the EM-FCTs’ validity levels. Theoretically, the results support a cognitive-based explanation for the FCT operation mechanism. Practically, they imply that FCT can be used in criminal or intelligence investigations of suspected members of terrorist or criminal organizations or suspected perpetrators of illegal acts or acts of terrorism, in which the incriminating evidence being sought is in the realm of designated semantic memory or knowledge.

## Introduction

The ability to detect lies and truths has crucial value in both criminal and intelligence investigations. Validating the veracity of an interviewee’s statement is an essential stage in preventing unlawful acts or acts of terrorism (or at least deciphering them retrospectively) and uncovering espionage actions (Evans et al., 2010). Therefore, the development and use of accurate, valid, and ethical methods for detecting lies of suspects, interviewees, intelligence sources, and sometimes even witnesses and victims are critical (Brandon et al., 2018).

In the last two decades, there has been a significant increase in the research and development of evidence-based methods and techniques for lie detection (Vrij et al., 2016). The most common research scenario simulates lying by commission or substitution. It requires the ‘experimental-lying’ participants to add or replace details of a specific event they experienced or to fabricate an entire report about an event that did not happen. For example, participants are asked to present an invented alibi for a time when they committed a mock crime or report attending a trip they did not actually attend (Vrij et al., 2018). Other research scenarios simulate lying by omission or concealment and test deception detection methods applicable to such settings as denying involvement in an investigated crime or omitting some details of it (Leal et al., 2020).

### The forced choice test

The deception detection forced choice type of test [also known as the symptoms validity test (SVT)] was initially developed for detecting malingering in a medical and neuropsychological context (Pankratz et al., 1975, 1987; Hiscock and Hiscock, 1989; Frederick et al., 1995). The method was further adapted to the forensic arena, aiming to detect the lies of suspects falsely claiming non-involvement in a crime under investigation and therefore no knowledge of its details (Shaw et al., 2014). The current paper focuses on this forensic application of the deception detection method, commonly presented as the forced choice test (FCT). The FCT addresses the deception-by-concealment forensic setting by including questions based on knowledge that only guilty suspects are expected to possess which is often called guilty or concealed knowledge. The test contains forced choice questions, usually with two optional answers: one correct and the other incorrect. The examinee is presented with the questions, one at a time, and requested to choose one of the answers or guess an answer. The underlying assumption of the FCT is that since innocent suspects do not know the answers to the questions but must, nonetheless, choose an answer, they will present a random answers pattern. An answers pattern with a significantly higher number of wrong answers than the expected random pattern indicates that the suspect is lying, i.e., they know the correct answers and intentionally avoid them (either because they are guilty of the crime or because they are innocent of the crime but were somehow exposed to guilty knowledge and are denying that).

Previous research on the FCT simulated real-life criminal and terrorism scenarios, such as denying participation in a burglary or in a terrorist act involving homemade explosives, concealing knowledge of terrorists’ hiding place, or concealing involvement in building a biological bomb (Morgan et al., 2014; Shaw et al., 2014; Orthey et al., 2018; Zhong and Kebbell, 2018). In general, findings showed that the FCT is a valid test able to distinguish between truth tellers and lie tellers (Orthey et al., 2017). Recent studies have examined other aspects of the FCT including developing other indicators for differentiating between truth tellers and lie tellers: for example, comparing performance in different parts of the test (Shaw et al., 2014) or testing response bias in a test that varies the strength of the differences between FCT stimuli (Orthey et al., 2019b); combining the FCT with other deception detection methods (Meijer et al., 2007; Nahari and Ben-Shakhar, 2011); or examining the test’s robustness against the training of suspects and against counter-measures (Verschuere et al., 2008; Orthey et al., 2018).

Although the FCT has been examined in many different scenarios, it has always simulated scenarios in which the lie tellers try to conceal their memory of details of a personally experienced event anchored in a specific time and place (Denney, 1996). Tulving (1983) defined such memory as episodic memory. He stated that episodic memory referred to knowledge about ‘temporally dated episodes or events, and temporal–spatial relations among these events’ and noted that such memory is stored ‘in terms of its autobiographical reference to the already existing contents of the episodic memory store’ (Tulving, 1972, p. 385). To the best of our knowledge, the FCT has never been examined in scenarios of the concealment of semantic memory—a memory of details and facts which are *not* grounded in personal experience. However, some critical criminal and terrorism-related scenarios are based on (the concealment of) semantic memory. Semantic memory is the ‘mental thesaurus, organized knowledge a person possesses about words and other verbal symbols, their meaning, and referents, about relations among them, and about the rules, formulas, and algorithms for the manipulation of these symbols, concepts, and relations’ (Tulving, 1972, p. 386). Semantic memory includes names (Lyons et al., 2002; Snowden et al., 2004), geographic locations (Viard et al., 2014; Davies and Tenbrink, 2018), and the verbal or conceptual descriptions of rules and regulations, procedures, and protocols (Tulving, 1972, p. 386; see also the reference to schemata or scripts in Tulving, 1983, p. 38). Some high-stakes criminal and terror-related investigations may involve lie tellers trying to conceal their affiliation and, therefore, their acquaintance with a criminal gang, a specific terror organization (Bohmer and Shuman, 2017), a hostile intelligence organization, or a particular commercial company (in the context of interstate espionage and industrial espionage). In such cases, lie tellers want to deny or conceal their knowledge of relevant details: for example, the names of the senior commanders or managers in the organization, the names and functions of its organizational units, its geographical sites, and its procedures and unique operational protocols. Other relevant and key semantic memory investigative scenarios focus on the concealment of general knowledge of criminal content worlds, such as weapons, illegal drugs trafficking, pedophilia, or money laundering, and of terrorism content worlds, such as the preparation of explosive devices or weapons training. Similarly, in such cases, lie tellers will want to conceal their knowledge of related procedures and modes of operations grounded in their semantic knowledge.

The current study tested the effectiveness of the FCT in detecting lies of semantic knowledge concealment, referred to here as semantic memory FCT (SM-FCT). We examined the SM-FCT using one type of semantic knowledge–knowledge of an organization—with the examinee either lying or telling the truth when denying being part of a criminal or terror organization. The study also compares the validity of the SM-FCT to the empirical validity values of the commonly examined episodic memory FCT (EM-FCT). This comparison was not conducted directly between two experimental groups with an experimental manipulation of the FCT type (SM-FCT, EM-FCT); rather, it was performed by comparing SM-FCT validity (and answers patterns) to previously found validity values in EM-FCT studies, as found in a benchmark we present below.

Although interrelated, semantic and episodic memory differ in processes such as encoding, storage, and retrieval as well as in their expression in amnesia and memory disorders. There is also evidence of different brain regions corresponding to different types of memories and clinical evidence of cases of differential damage to only one type of memory (Duff et al., 2020; Coane et al., 2021). This leads to the question of how the type of memory involved might interact with FCT effectiveness.

Several theoretical explanations have been proposed in recent years for the effectiveness of the FCT as a deception detection technique. Some theoretical explanations have focused on motivational-emotional aspects. These explanations are based on the strategic avoidance theory (Shaw et al., 2014) which attributes to lie tellers a tendency to avoid any information on the subject about which they are being investigated (Hartwig et al., 2005; Granhag et al., 2013). Semantic memory lacks the highly personal, emotional, and motivational aspects that characterize episodic memory (Damasio, 1994; Davidson, 2003). This semantic memory deficiency may reduce the lie teller’s propensity to avoid familiar knowledge (Allen et al., 2008; Shaw et al., 2014), thus reducing the ability of the SM-FCT to differentiate between truth tellers and lie tellers and lowering its validity compared to the EM-FCT. The SM-FCT lower validity hypothesis can also be derived from studies showing that the more prevalent the lie is in one’s in-group (Gino et al., 2009) and the more significantly it helps the in-group (Gino et al., 2013), the higher the likelihood that people will choose to perform it and even regard it as less severe. Concealing semantic knowledge, such as affiliation with a terrorist organization, would be a lie that all members of this organization would be expected to tell and that would benefit all. Consequently, such lies will be perceived as less negative in value and thus less threatening in the SM-FCT scenario, leading lie tellers to feel less of a need to avoid choosing answers that link them to this lie. Consider, for example, a suspect affiliated with a terrorist organization when asked an FCT question about general procedures and characteristics of a typical illegal act (e.g., vandalizing) conducted occasionally by most of the organization’s members (semantic memory). Such a suspect will feel less of an extreme need to avoid choosing the correct answer – i.e., less of a need to avoid a response of partially admitting their connection with these illegal acts – because they know that many people in their environment conduct these illegal acts, and many benefit from it. However, this will not be the case when such acts are conducted only by the suspect and only for their own benefit (episodic memory). This, in turn, may reduce the ability of the SM-FCT to detect lie tellers. In sum, the motivational-emotional explanation of the deceptive examinee’s performance in the FCT entails the prediction that the SM-FCT will have a lower validity than the EM-FCT.

Other explanations for the validity of the FCT as a deception detection technique have relied on cognitive theories (Meijer et al., 2007) that emphasized the lack of understanding of probabilistic and random processes (Falk and Konold, 1997). This cognitive-oriented FCT validity conceptualization (Meijer et al., 2007) states that the primary mechanism behind the success of the FCT is the failure of lie teller examinees to produce a series of answers that resemble a random series typical of guesswork. If that is the case, then the SM-FCT and EM-FCT should not differ in their validity, since the need to produce random-like answers is the same for both testing scenarios. This prediction can also be supported by the successful implementation of the cognitive hierarchy theory (CHT; Camerer et al., 2004) to explain FCT examinees’ performance (Orthey et al., 2017). Orthey et al. (2017) demonstrated that the cognitive selection of strategy provides a better theoretical framework for deceptive examinees’ behavior in the FCT than mere avoidance motivation. In sum, the cognitive explanation for deceptive examinees’ performance in the FCT predicts that the SM-FCT will have similar validity to the EM-FCT since the cognitive challenge to produce a random pattern of correct and incorrect answers is the same in both scenarios.

The current study asserts that it is possible to weigh the two explanations comparatively and thus reach a prediction that favors one against the other. The FCT is a type of guilty knowledge test (GKT) for deception detection. When taking the FCT, the examinees must choose a correct or incorrect answer to each consecutive question. They try to answer the questions in a way that presents them as not possessing any relevant knowledge and therefore not connected to the issue under investigation. This study therefore conceptualizes the FCT examinees’ performance as reflecting, primarily, conscious and repeated decision-making processes [cf. the polygraph GKT which is based on automatic and autonomous responses to emotionally arousing stimuli (see Nahari and Ben-Shakhar, 2011)]. We thus assume that: (1) the prominent factor responsible for lie tellers’ performance pattern (which enables their exposure via the FCT) is a flawed and cognitively-biased decision-making process that reflects a misconception of random patterns and leads to an excessive number of wrong answers; and (2) this flawed and cognitively-biased decision-making process factor outweighs other possible factors.

The current study makes it possible to test empirically and comparably the two categories of explanations underlying the FCT. Lower validity of the SM-FCT compared to the validity values found previously in studies that tested the EM-FCT supports the motivational-emotional explanations that underlie the personal avoidance response of the lie teller. Similar SM-FCT and EM-FCT validity supports explanations based on the cognitive ability to produce random series and the difficulty thereof. As presented above, based on the literature review, we assume that the primary mechanism behind the validity of the FCT is not the type of memory concealed (episodic or semantic) but rather the lie tellers’ inability to produce the random-like answers pattern typical of guesswork. We therefore predicted that the SM-FCT test would be valid and its validity would be within the value range of the validity levels of EM-FCT obtained in previous studies. That is, we predicted that the validity of SM-FCT will be non-inferior to the lowest level of validity obtained in previous EF-FCT studies. This prediction is included in the current study’s preregistration.^1^

To specify a hypothesis comparing the validity of the SM-FCT to the previously found validity values in EM-FCT studies demands a benchmark of such studies. Seven studies thus far have examined the validity of a two-alternative EM-FCT in a forensic, mock crime context with naïve (untrained) participants and reported the test’s sensitivity in detecting lie tellers (within the accepted cut-off point of 95% specificity while using the number of correct answers as a veracity indicator) [Merckelbach et al., 2002; Jelicic et al., 2004; Meijer et al., 2007 (Study 1); Giger et al., 2010; Orthey et al., 2017, 2018 (only naïve/untrained participants); Orthey et al., 2019a (no time pressure group)]. Sensitivity measures ranged between 27 and 59%. We hypothesized that the sensitivity of the SM-FCT will not be inferior to that of the EM-FCT when using the number of correct answers veracity indicator. The predicted lower limit sensitivity value of the SM-FCT used in this hypothesis was therefore set to the minimum sensitivity value of 27% found among all of the seven studies included in the aforementioned benchmark. Therefore, the study’s first hypothesis (H1) stated that the SM-FCT sensitivity using the number of correct answers veracity indicator will not be inferior to 27%.

Two of the aforementioned seven studies also included the use of a follow-up runs test for the participants who performed within the random range of the number of correct answers as a way of increasing the test’s sensitivity (Merckelbach et al., 2002; Jelicic et al., 2004). A run is either a sequence of adjacent correct answers or a sequence of adjacent incorrect answers (Verschuere et al., 2008). Thus, in the sequence ‘incorrect, incorrect, incorrect, incorrect’ the number of runs equals one, and in the sequence ‘incorrect, incorrect, correct, incorrect, correct, correct’ the number of runs equals four (first run: ‘incorrect, incorrect’; second run: ‘correct’; third run: ‘incorrect’; fourth run: ‘correct, correct’). Some lie tellers may try to aim for an equal number of correct and incorrect answers, looking to represent the ‘probability = 0.5’ aspect of a random pattern (Jelicic et al., 2004). They may decide that the simpler and less cognitively demanding way of doing so is to start with either a correct or an incorrect answer and then continue alternating between correct and incorrect answers for all or most of the questionnaire (i.e., an answer pattern of: ‘correct, incorrect, correct, incorrect, etc.’), particularly as they do not know the total number of questions. This may lead to a higher number of runs than the number expected from a random pattern. In contrast to the pattern of lie tellers mentioned above, other lie tellers may start with many incorrect answers and then, at some point in the middle of the sequence of questions, realize that this strategy may indicate their knowledge of the correct answers and thus expose them as lie tellers. At that point, they may try to ‘compensate’ for their former abundance of incorrect answers by choosing an excessive number of correct numbers. This pattern of answers may lead to a lower number of runs than the number expected from a random pattern. However, following this rationale and using the number of runs and its significant deviation from a random pattern as an additive veracity indicator, none of the tested participants in Jelicic et al.’s (2004) and in Merckelbach et al. (2002) studies, performed outside of the random range in the runs test, amounting to a 0% sensitivity increase. Since in some non-mock crime studies a positive runs-based increased sensitivity was found (e.g., Verschuere et al., 2008), the study’s second hypothesis (H2) stated that the SM-FCT increased sensitivity, using the follow-up runs test veracity indicator, will also be positive.

On the group level, we predicted that the deceptive participants’ number of correct answers will be lower than expected by the binomial distribution (of *p* = 0.5) and the number of runs within that group would be higher than expected by that distribution (H3 and H4). Some existing FCT studies showed that, in addition to a significantly high number of participants producing a lower number of correct answers than expected by the binomial distribution, there is also a significantly high number of participants producing a higher number of correct answers than expected by the binomial distribution (Orthey et al., 2017). This phenomenon may lead to a prediction that deceptive participants will vary more in their number of correct answers than the expected binomial distribution variance (H5).

The current study tested the following hypotheses regarding the SM-FCT: (H1) the sensitivity in detecting lie tellers using the number of correct answers indicator will not be lower than 27% (the lowest value of sensitivity in the EM-FCT studies’ benchmark mentioned above); (H2) the SM-FCT increased sensitivity, using the follow-up runs test veracity indicator, will be positive; (H3) lie tellers will produce a lower percentage of correct answers than expected by a random answers pattern (50%); (H4) lie tellers will produce a higher percentage of runs than expected by a random answers pattern (50%); (H5) the lie tellers’ dispersion of correct answers will be higher than expected by a random answers pattern. All hypotheses, apart from H2, were preregistered on the Open Science Framework (OSF) (see text footnote 1).

## Materials and methods

### Participants

Participants were 286 first-year psychology undergraduate students at an academic college in Israel who participated in the study as part of their course requirements. Our target preregistered sample size of *n* = 284 was based on assumed effect size of an expected sensitivity level of 0.46 (46%) for the SM-FCT—derived from the average sensitivity obtained in the studies reviewed in the aforementioned benchmark. With alpha = 0.05 and power of 0.85, a minimum sample size of 26 participants was required for a non-inferiority test in which we tested the data against P0 = 0.27 and non-inferiority margin of 5% or 0.05. The computation was conducted using G*power software (Faul et al., 2007). The current study was a part of a wider project; thus, the actual target preregistered sample was *n* = 284 (for details, see text footnote 1). Nine participants were excluded from the study due mainly to violation of the standard testing conditions (e.g., problems with the video conference call, too much background noise) or problems understanding the simulation instructions. The final sample included 277 participants (82% women; age: *M* = 23.53 years, *SD* = 1.68).

### The SM-FCT questionnaire

The current study simulated a scenario of interviewing people suspected of being members of a criminal or terror organization in an attempt to detect lie tellers falsely denying this. We simulated this scenario by asking students to falsely deny being part of the college where they actually study. The initial set of questions was built by the researchers and a group of six research assistants who were third-year students in the college. This group effort yielded an initial set of 115 questions. The questions were about different semantic knowledge facts pertaining to the students’ affiliation to the college. They included questions about names (e.g., ‘What is the name of the president of the college: Lisa Fischer or Jane Ryan?’); geographic locations (‘Which of the following serves as a meeting place for the students during breaks: the lawn in front of the computer building or the lawn in front of the business administration building?’); and college or academic program rules and regulations (‘Entrance to the college campus requires a parking permit affixed to the windshield or presentation of a student card?’; ‘How many mandatory courses in sociology is a student required to take in the first semester of the first year: one or two?’). None of the questions were about a specific event anchored in a particular time and place (e.g., ‘Which band performed at the opening ceremony of the current academic year?’).

Two pilot tests were conducted before finalizing the FCT. Pilot A aimed to confirm that the likelihood of choosing either of the two answers to each question would be similar for people not affiliated with the college (Doob and Kirshenbaum, 1973). This pilot stage is common in FCT studies. A group of 20 participants was recruited using convenience sampling from the general population (i.e., not students at the college) and asked to complete the questionnaire. Only the questions whose correct answer was chosen by 30–70% of respondents were retained (Doob and Kirshenbaum, 1973), which led to 24 questions being omitted at this stage. Pilot B aimed to confirm that the correct answers were known to people affiliated with the college. This second condition has not been tested as a pilot stage within the construction process of previous FCT studies, but we introduced it since any question whose answer is not known by most guilty examinees may decrease the test validity. A different group of 20 students studying in the college completed the Pilot B revised questionnaire as a knowledge test. These students were sampled from the same population as the participants of the main study. Only questions whose correct answer was chosen by 80%^2^ of the respondents or more were retained, which led to another 45 questions being omitted at this stage. Following these two pilot tests, the final version of the FCT included 46 questions, each having two possible answers (correct and incorrect), being reasonably balanced (Pilot A), and being known to the lie tellers to an acceptable degree (Pilot B). The position of the correct answers between the 1st and 2nd alternatives presented was randomized when constructing the FCT suspect. All participants received the questions in the same order.

### FCT scoring

#### Number of correct answers

For each correct answer selected, participants scored one point. Over the 46 questions, participants could therefore score between 0 (all incorrect answers selected) and 46 (all correct answers selected).

#### Number of runs

Previous studies (e.g., Verschuere et al., 2008) used the number of runs as another FCT measure (see above). In a 46-questions FCT, the number of runs varies between 1 and 46.

#### Design and data analysis

For a detailed description of the study design and data analysis, including the calculation resulting in the decision rule that participants who answered 16 questions or fewer correctly would be defined as lie tellers, see Appendix A.

### Procedure

Participants were invited by email to take part in an experiment in which they ‘try to trick an expert in deception.’ They were told that if they succeeded in deceiving the expert, they would be entered into a raffle with a NIS 500 (about $150) prize. Due to COVID-19 social distancing restrictions, the study was conducted remotely using the Zoom communication platform and the study sessions were video recorded. Participants signed an informed consent form. Interviewers sat in front of a computer with a white background behind them.

Upon entering the Zoom session, the participants were greeted by a member of the research team who documented their age and gender. Participants were asked to grade their motivation to succeed in the interview on a 5-point Likert-type scale from 1, ‘not at all motivated to succeed,’ to 5, ‘highly motivated to succeed.’ Participants were then told that a serious crime has been committed in their college and that the police have information indicating that the perpetrator studies in their college and are trying to find out which of the participants are studying at the college and which are not. They were told that, in a second interview which would start immediately, they must lie to the interviewer and deny studying at the college in question. They were also told that the interviewer will not know whether they are telling the truth or lying but only that some of the participants are telling the truth (i.e., they do not study at the college as they will claim) and some lying (i.e., they do study at the college but will falsely deny it) and that they will therefore be suspicious from the outset. (In fact, all participants were lie tellers, which happens frequently in FCT research [see, for example, Verschuere et al., 2008; Orthey et al., 2019a)]. Participants were told that if the interviewer believes them, they will be entered into a raffle with a prize of NIS 500; if, however, the interviewer does not believe them, they will not be entered into the raffle and will, instead, be required to complete a tedious writing task.

Each participant then entered a Zoom session with the interviewer who, indeed, told them they were investigating a crime committed at the college and known to have been committed by a student at the college. The interviewer explained that they were therefore looking to find out which of the participants study at the college. Those found affiliated with the college will, they stated, have to undergo additional interviews and write a detailed report describing their actions over the past week; those found not affiliated with the college will have to undergo no further procedures after this interview.

After this introduction, the interviewer asked the participant whether they studied at the college in question and confirmed their negative answer. The interviewer then explained that they will ask several questions about the college—each with two possible answers—and the participant must choose one answer according to their knowledge about the college or guess the answer if they have no prior knowledge. The interviewer then read each question and the two possible answers and documented the answer chosen by the participant. At the end of the session, a debriefing process was performed and the participant was asked not to disclose any details to other potential participants. Contrary to the original explanation, all participants were entered into the raffle and no one was asked to complete the writing task. To properly compare the SM-FCT and EM-FCT, three other test delivery variations were used representing the different ways EM-FCT was examined in previous studies. For a description of these three other test delivery variations, see the design and analysis plan that were preregistered at the OSF (see text footnote 1). Analysis of the different outcomes of these variations is beyond the scope of the current paper.

This research was approved by the Institutional Review Board (IRB) of the Academic College of Tel Aviv-Yaffo (# 2020193/58). The study’s design, analysis plan, and hypotheses were preregistered at the OSF and can be accessed at https://osf.io/sa8gq, where we have also reported how we determined our sample size, all data exclusions, all manipulations, and all measures used. All data and the study materials have been made publicly available at the OSF and can be accessed at https://osf.io/rtgk8/?view_only=d87fb652a2904bb99bd84f60e57f6f83.

## Results

### Manipulation check

Of all the participants, 98% (270/277) ranked their motivation to succeed in the interview as 4 or 5, which was above mid-scale (value of 3 in a 5-point Likert-type scale). This finding confirmed that the vast majority were motivated and committed to making an effort to succeed in the experimental simulation.

### Hypothesis testing

#### Analysis of H1: the sensitivity in detecting lie tellers will not be lower than 27%, with non-inferiority margin of 5% or 0.05

As described above, participants who answered 16 questions or fewer correctly were defined as lie tellers. Using this cut-off point, the sensitivity of the SM-FCT totalled 31.0% (86/277): that is, 31.0% of the lie tellers scored below the pre-defined cut-off point (below chance), answering correctly 16 questions or fewer. In accordance with H1, our analysis found evidence for non-inferiority of the sensitivity of the SM-FCT relative to the EM-FCT. A one-sample z-test for proportion found that the SM-FCT sensitivity (31.0, 95% one-sided lower limit CI = [26.5%, ∞]) had a lower bound that was indeed higher than the lowest value of the EM-FCT sensitivity (27%) within the pre-determined non-inferiority margin of 5% (22%), *z* = 3.56, *p* < 0.001, *h* = 0.21, 95% CI = [0.09, 0.33]. Assuming an equal number of truth tellers and lie tellers, the overall accuracy (balanced accuracy) of the SM-FCT, computed as the average of a test’s sensitivity (31.0%) and specificity (95%), amounted to 63%. H1 was thus supported.

**Figure 1:**
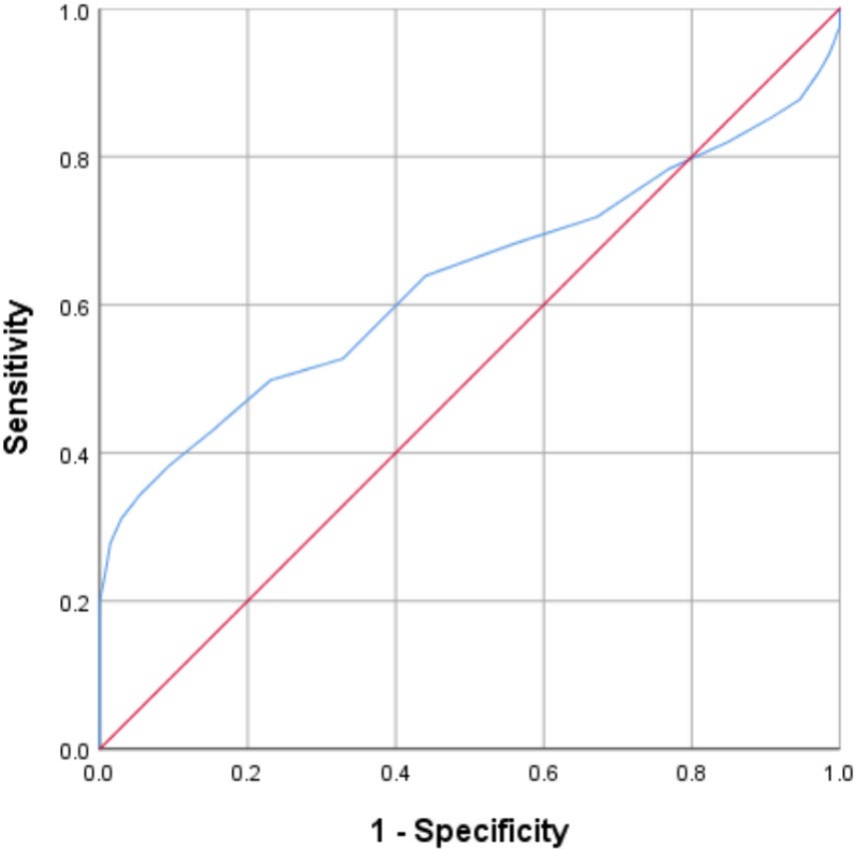
ROC curves for the validity of the FCT’s number of correct answers indicator.

Figure 1 displays the ROC plot depicting the sensitivity against 1 – specificity for all possible cut-off points using the main FCT indicator: number of correct answers.

As seen in Figure 1, a low number of correct answers differentiated lie tellers from truth tellers better than chance, AUC = 0.63, *p* < 0.001, 95% CI [0.58, 0.68].

#### Analysis of H2: the SM-FCT increased sensitivity, using the follow-up runs test veracity indicator, will be positive

As described above, we defined participants as lie tellers when they did not meet the first decision rule (defined by the number of correct answers) but presented sequences with fewer than 18 or more than 28 runs. Our analysis found that 28 participants who did not meet the first decision rule did meet the follow-up sequences decision rule. This finding suggests that the increase in the sensitivity in detecting lie tellers using the follow-up runs test amounted to 10% (28/277), (95% one-sided lower limit CI = [7.1%, ∞])—resulting in an increase from 31% sensitivity of the SM-FCT to 41% sensitivity of the SM-FCT—which supports the hypothesized positive increased sensitivity. H2 was thus supported.

#### Analysis of H3: lie tellers will produce a lower percentage of correct answers than expected by a random answers pattern (50%)

A one-sample z-test for mean found that the number of correct answers chosen by the lie tellers (*M* = 19.88, 95% CI = [19.48, 20.28]) was indeed lower than the expected value of 23 (*p* = 0.5 or 50% out of the total 46 questions), *z* = −15.31, *p* < 0.001, *d* = 0.92, 95% CI = [0.78, 1.06]. H3 was thus supported. In terms of proportions or percentage, the sample mean equaled *p* = 0.43 or 43% of the total number of questions which is 46; the respective CI for the proportion of correct answers was = [0.426, 0.434].

#### Analysis of H4: lie tellers will produce a higher percentage of runs than expected by a random answers pattern (50%)

In accordance with the analysis of H2, we tested H4 on a subgroup of the 53 participants whose number of correct answers was 22–24 (the value of number of correct answers expected by a random answers pattern [23] plus or minus 1). A one-sample z-test for mean found that the number of runs made by lie tellers (*M* = 25.75, 95% CI = [24.85, 26.65]) was indeed higher than the expected value of 23.5 (*p* = 0.5 or 50% out of the possible range of 1–46 questions), *z* = 4.88, *p* < 0.001, *d* = 0.67, 95% CI = [0.37, 0.97]. We also conducted the same analysis with a subgroup of the 12 participants whose number of correct answers was 23 (the exact value of number of correct answers expected by a random answers pattern). Results followed the same pattern (number of runs was significantly higher than expected by chance). H4 was thus supported.

#### Analysis of H5: the lie tellers’ dispersion of correct answers will be higher than expected by a random answers pattern

Our analysis found a sample variance estimator of (*s^2^* = 56.85, with Bias = −0.176, and a 95% CI = [48.93, 64.99]), which was indeed higher than the expected value of 11.5 (K*50%*50%). H5 was thus supported.

## Discussion

The SM-FCT produced differentiating answers patterns between lie tellers and truth tellers^3^, similar to those found for the EM-FCT in previous studies; all the research hypotheses were thus supported. Consequently, the SM-FCT was shown to have a similar and non-inferior validity in deception detection compared to the range of the EM-FCT validity levels. These results were demonstrated in the following ways.

First, the study found that the number and percentage of correct answers chosen by lie tellers who try to conceal their semantic knowledge is lower than expected by a random answers pattern. Second, the sensitivity of the FCT demonstrated in the semantic memory scenario (SM-FCT) was found to be about 30%, which is within the range of sensitivities values measured in episodic memory studies (EM-FCT) and not inferior to the lower limit found in such studies. This finding was echoed in the ROC curve (see Figure 1) with an AUC of the number of correct answers indicator suggesting above chance accuracy levels and having a similar value to the ones found in previous studies (e.g., Orthey et al., 2019a). Third, the variance of the correct answers within the lie tellers’ group in SM-FCT was higher than the variance expected by a random answers pattern. This is another aspect of the similarity between SM-FCT and EM-FCT. Fourth, as a group, the lie tellers who answered the SM-FCT with a similar number of correct answers to that expected by a random pattern produced sequences of answers with a higher number of runs than expected by the same random pattern. Finally, this last finding was followed by a positive increased sensitivity achieved by using the follow-up runs test in the SM-FCT (about 10% addition to the sensitivity with only the number of correct answers indicator).

The similarity between the lie tellers’ answers patterns and test validity found in the SM-FCT and those found in the previously investigated EM-FCT lend support to the hypothesis that the FCT is robust in the transition from episodic to semantic memory scenarios. This robustness has both theoretical and practical implications. Theoretically, these results support the explanations for an FCT operation mechanism based on cognitive ability and the difficulty of producing random series of answers in FCT (Meijer et al., 2007; Orthey et al., 2017) over the motivational-emotional factors that may underlie the personal avoidance responses of lie tellers (Shaw et al., 2014). Practically, these results imply that the FCT can be used in investigations concerning criminal or intelligence scenarios with potential suspects of illegal acts or acts of terrorism, where the incriminating evidence the authorities are looking for is in the realm of specific semantic memory or knowledge. Specifically, the study results support the possibility of using forensics FCT in intelligence gathering and investigation to identify whether a suspect who denies affiliation with a terrorist organization is telling the truth or lying in their denial. This may be achieved by detecting their potential concealments of relevant semantic memory, such as a terrorist organization’s commanders’ identity, and procedures, geographical aspects of its activity, and more.

In addition to testing the FCT in semantic memory scenarios, the current study presented two novel elements within the structuring and administrating of the FCT. First, in constructing the test items, we included a knowledge test as a preceding pilot procedure for the test items. By using the pilot’s outcome, we were able to omit test questions based on specific pieces of information which, contrary to the assumptions of the test creators, were not possessed by people holding the relevant semantic knowledge. While this pilot test is sometimes used after the FCT administration, it appears to have never been used before the test’s administration and as part of its construction stages. The more test items there are which are not known to the lie tellers, the higher the epistemological similarity between them and the truth tellers. Consequently, the potential differences between lie tellers and truth tellers expected to emerge in the FCT answers pattern decreases, which impedes the test’s sensitivity. We recommend using this procedure regularly as a pilot test alongside the more well-known pilot test that examines the degree of balance between the plausibility levels of the various answers (Doob and Kirshenbaum, 1973).

We acknowledge that this recommendation is not easy to fully implement in real-world investigations; however, several proxy options could be used. For example, in the case of a bank robbery, one could have innocent people visit the same bank at a similar time and then, afterwards, asked what they remember and what they do not (e.g., the color of the bank teller’s shirt). In other cases of an ongoing investigation, one could find and interview only some of those involved in the first stage. If they cooperate, they could be asked what they remember from the event and what they do not and the FCT questionnaire could be updated accordingly (they may even provide new details that allow the addition of new questions to the original FCT questionnaire). Finally, when using an FCT that tests affiliation with an organization, one could check what a member of that organization really does know (in case they serve as an intelligence source) and determine accordingly whether certain questions should be included in the FCT questionnaire or not.

The second novelty of this study was the use of long-distance administration of the FCT via Zoom communication. As in many research projects conducted during the COVID-19 pandemic, this was due more to the social distancing constraints than being one of the research goals. Nevertheless, as a by-product of this necessity, it can be cautiously stated that the Zoom test administration does not seem to impair the ability of the FCT to detect lies. Further systematic research is, however, needed to test this issue.

The study has three main limitations. First, and similar to the vast majority of deception detection research, the study was lab-based with participants instructed to lie (rather than choosing that option) by presenting themselves as not possessing mundane knowledge of details concerning an academic institute (rather than terror-related knowledge) and with limited stakes for the participants. Recent research has suggested that there are unlikely to be significant differences between the validity levels of the tools for detecting lies as measured in the laboratory and the validity level of tools in field use or tools using directed lies in contrast to spontaneous lies (Geven et al., 2018). Nonetheless, we recommend conducting field studies on FCT, as we are unaware of any such studies previously conducted. Second, and more specific to our research objectives, we tested the validity of the SM-FCT on only one type of semantic knowledge: knowledge about organizations. Although we cannot think of any theoretical reason why testing the FCT in a different sematic memory scenario would yield different results, we still recommend examining the FCT with other types of semantic knowledge, such as knowledge of the protocol of action (e.g., assembling explosive devices) or knowledge concerning general issues (e.g., drugs or weapons). Third, as mentioned above, we assume that using Pilot B in constructing the FCT and, consequently, omitting questions that are found to rely on knowledge that lie tellers do not have, increases the test’s sensitivity. Despite being an improved test construction procedure, it may limit somewhat the ability to compare the SM-FCT sensitivity achieved in the current study to the sensitivity values of previous EM-FCT studies, as this pilot was conducted in the current study but not in the previous ones. Future studies could compare SM-FCT and EM-FCT sensitivity values using the same pilot stages.

The current study contributes more generally to the relationship between science and practice by supplying an additional opportunity for practitioners to make use of an evidence-based method. It thus joins the current constructive and beneficial trend of knowledge exchange between researchers and practitioners, which is greatly needed by both scientists and law enforcement and intelligence agencies (Russano et al., 2019). This study addressed the distinction between the SM-FCT and the EM-FCT, and its findings support the hypothesis that the FCT is a robust test across different types of memories. While the extent of the test’s robustness regarding other applications, scenarios, and events types remains a question to be addressed in future studies, our study, alongside former studies showing FCT validity, serves as a recommendation for practitioners to make operational use of this valid and evidence-based deception detection method.

## Data availability statement

The datasets presented in this study can be found in online repositories. The names of the repository/repositories and accession number(s) can be found at: https://osf.io/rtgk8/?view_only=d87fb652a2904bb99bd84f60e57f6f83.

## Ethics statement

The studies involving humans were approved by the Institutional Review Board (IRB) of the Academic College of Tel Aviv-Yaffo (# 2020193/58). The studies were conducted in accordance with the local legislation and institutional requirements. The participants provided their written informed consent to participate in this study.

## Author contributions

TA: Conceptualization, Data curation, Formal analysis, Funding acquisition, Investigation, Methodology, Project administration, Resources, Supervision, Writing – original draft, Writing – review & editing. GG: Data curation, Project administration, Supervision, Writing – original draft, Writing – review & editing. AV: Writing – review & editing. SL: Writing – review & editing.

## Footnotes

^1^ https://osf.io/sa8gq

^2^Since using a knowledge test of the correct answers as a pilot in the construction of the FCT was done for the first time in the present study, we had no previous empirical reference to rely on in this context. In two earlier studies where this knowledge test was used (Orthey et al., 2019a, 2022), the percentage of correct answers was 91.2 and 91.5, respectively. Assuming an equal distribution of the number of correct answers, using the threshold chosen in the current study—questions in which a minimum of 80% and a maximum of 100% of the participants answer correctly—the expected percentage of correct answers should amount to about 90%, consistent with the value found in these previous studies. Another study that examined this issue (Nahari and Ben-Shakhar, 2011) found that the percentage of correct answers in participants tested immediately after receiving the information was 86%, also compatible with the threshold chosen in the current study.

^3^The truth tellers’ answers pattern used for that comparison is the assumed random answers pattern of a guessing truth teller examinee.
