# Supplementary material for: Successful use of the forced choice test for detecting concealment of semantic memory in criminal and intelligence investigations
Source: Front Psychol. 2024 Jun 7;15:1399985. doi: 10.3389/fpsyg.2024.1399985 (PMC11192207; doi:10.3389/fpsyg.2024.1399985)
Supplement: Supplementary file 1 [file Data_Sheet_1.docx]

APPENDIX A: Detailed Description of the Study Design and Data Analysis

In the FCT test, it is customary to determine a decision rule for identifying lie tellers according to the degree of the statistical deviation of their answers pattern relative to the theoretical binomial distribution assumed for the truth tellers. Therefore, many studies that aim to test the validity of the FCT (e.g., Orthey, Palena, et al., 2019; Verschuere et al., 2008) do not include participants who play the role of truth tellers but only those who play the role of lie tellers. Instead of comparing the lie tellers’ answers pattern to a different experimental group of truth tellers, they compare their answers pattern to the assumed random answers pattern of truth tellers (as also being confirmed by the results of Pilot A mentioned above). The level of the FCT’s accuracy in identifying the lie tellers is established by the proportion of lie tellers who perform higher than the FCT pre-defined cut-off point (sensitivity). This cut-off point is determined according to a value that will identify 95% of the truth tellers as such (95% specificity), amounting to 95% FCT accuracy for the truth tellers. This data analysis enables the testing of the two-fold accuracy of the FCT and, consequently, the existence of a difference between truth tellers and lie tellers in a study design that includes only participants playing the role of the latter. In light of this, all the participants in the current study also played the role of lie tellers.

For H1, Siegel’s (1956) binomial formula was used to determine the z-score for the FCT’s number of correct answers: z = ((x ± 0.5) − NP/√ (NP (1 − P)). Following common practice in FCT research, we used a z-score cut-off of z < − 1.65 (one-tailed, significant at p = .05, with the standard pre-determined specificity of 95%), meaning that those who answered 16 questions or fewer correctly would be defined as lie tellers. The test sensitivity was defined as the proportion of lie tellers who were detected as such according to the aforementioned number of correct answers indicator. In order to test the hypothesis that this sensitivity will not be lower than 27%, we conducted a non-inferiority test (Lakens et al., 2018) with the pre-determined and preregistered on the OSF ([https://osf.io/sa8gq](about:blank)) non-inferiority margin of 5% or 0.05. This 5% non-inferiority margin was based on personal communication with field agents who stated that differences above but not below such value are usually considered significant for the accuracy or validity of a deception detection method.

We assumed that truth tellers would show random answers pattern. Therefore, similar to Orthey, Vrij, et al. (2019), we compared our distribution of lie tellers to a random distribution representing the answer pattern of truth tellers. However, in contrast to Orthey, Vrij, et al. (2019) who used a computer simulation of a random answers pattern, we used the expected values of the binomial distribution as a theoretical simulation of the random answers pattern, as truth tellers’ performance is represented by that distribution. We focused on reporting the validity of the FCT using its main indicator: the number of correct answers. Therefore, we constructed a 277 (similar *n* for comparative reasons) theoretically-based truth tellers comparison group with the expected statistical characteristics of the aforementioned binomial distribution (*p*=0.5, *n*=46) (e.g., one truth teller with 14 questions answered correctly, two truth tellers with 15 questions answered correctly, four truth tellers with 16 questions answered correctly, etc.^[[1]](#footnote-1)^). As a detection performance measure, we used the signal detection theory parameter of area under the curve (AUC) of the receiver operating characteristic (ROC). The ROC plots the correct detection rate for lie tellers (sensitivity) against 1 – the correct detection rate for truth tellers (1 – specificity) for all possible cut-off points. The AUC measure represents the detection accuracy across all possible cut-off points, serving as a general measure of detection accuracy for the entire scale, independent of any specific cut‐off points. The AUC ranges from 0 to 1, with 0.5 indicating chance performance. Values significantly higher than 0.5 suggest that the criterion has diagnostic value. The probabilistic interpretation of the AUC empirical value in the current scenario is the average probability of a randomly selected truth teller answering more correct answers than a randomly selected lie tellers, across all pairs of truth tellers and lie tellers (for more details on signal detection theory and the AUC see Wickens [2001]).

For H2, Siegel’s (1956) binomial formula was also used to determine the z-score for the FCT’s number of runs indicator, as this measure follows the same binomial distribution (except for having a minimum value of 1). We followed the aforementioned Verschuere et al.’s (2008) decision rule for considering both higher and lower number runs as indications of deception. We computed a z-statistic, as described above. Accordingly, sequences with fewer than 18 or more than 28 runs deviate significantly from chance at p = 0.05. The test increased sensitivity using the follow-up runs test was defined as the additional proportion of lie tellers who were not detected as lie tellers using the number of correct answers indicator but were detected as such using the follow-up runs test. We tested whether that increased sensitivity was positive.

H3 and H4 concerned the number of correct answers and the number of runs at the group level. H3 predicted that the lie tellers’ group average number of correct answers would be smaller than *M* = 23 (less than 50% of the number of questions). H4 predicted that the lie tellers’ group average number of runs would be greater than *M* = 23.5 (less than 50% of the number of runs which, as mentioned below, varies between 1 and 46). Both hypotheses compare the FCT lie tellers’ performance against H0, characterised by the expected hypothetical binomial distribution of B(n=46, p=0.5).^[[2]](#footnote-2)^ The H0 population variances are therefore known to be: Var = n*p*q = 11.5. Consequently, the sampling distribution is a z-normal one. Therefore, in order to test H3 and H4 we conducted a one-sample z-test for mean (with known population variance) and computed confidence intervals accordingly. Corresponding with the analysis of H2, we tested H4 on a subgroup of the participants whose number of correct answers was very close to that expected of a random pattern.

H5 predicted that the group variance of the FCT lie tellers’ number of correct answers would be higher than the expected hypothetical binomial distribution of B(n=46, p=0.5) variance, which, as mentioned above, is Var = n*p*q = 11.50. The chi-square test commonly used for testing hypotheses about a single variance of a population is not robust against departures from normality (Long & Sa, 2005). Since the distribution of the lie tellers’ number of correct answers is assumed to be non-normal (Orthey et al., 2017), we used the bias-corrected and accelerated bootstrap method for constructing a 95% confidence interval for the population variance of the number of correct answers chosen by lie tellers (Efron, 1987; Puth, et al., 2015).

1. Due to rounding integers for each expected number of truth tellers answering each specific number of questions correctly, the total number of the truth tellers comparison group did not add up to 277 (but to 274). We therefore added one expected truth teller with 13 questions answered correctly, one with 23 questions answered correctly, and one with 33 questions answered correctly. [↑](#footnote-ref-1)
2. Strictly speaking, the number of runs does not have a binomial distribution since its minimum value is 1 and not 0. In order to solve that problem, we used the number of switches (number of runs minus one), which varies between 0 and 45 and therefore does have a binomial distribution. We then transformed the results back into the number of runs in order to compare with previous studies. [↑](#footnote-ref-2)
